# Extent of arterial calcification by conventional vitamin K antagonist treatment

**DOI:** 10.1371/journal.pone.0241450

**Published:** 2020-10-29

**Authors:** Selma Hasific, Kristian Altern Øvrehus, Oke Gerke, Jesper Hallas, Martin Busk, Jess Lambrechtsen, Grazina Urbonaviciene, Niels Peter Rønnow Sand, Jens Steen Nielsen, Louise Diederichsen, Kenneth Bruun Pedersen, Rasmus Carter-Storch, Nivethitha Ilangkovan, Hans Mickley, Lars Melholt Rasmussen, Jes Sandal Lindholt, Axel Diederichsen

**Affiliations:** 1 Department of Cardiology, Odense University Hospital, Odense, Denmark; 2 Department of Nuclear Medicine, Odense University Hospital, Odense, Denmark; 3 Clinical Pharmacology and Pharmacy, University of Southern Denmark, Odense, Denmark; 4 Department of Cardiology, Little Belt Hospital Vejle, Vejle, Denmark; 5 Department of Cardiology, Odense University Hospital Svendborg, Svendborg, Denmark; 6 Department of Cardiology, Regional Hospital Central Jutland Silkeborg, Silkeborg, Denmark; 7 Department of Cardiology, Hospital South West Jutland Esbjerg, Esbjerg, Denmark; 8 DD2, Steno Diabetes Centre Odense, Odense University Hospital, Odense, Denmark; 9 OPEN, Odense Patient Data Explorative Network, Odense University Hospital, Odense, Denmark; 10 Department of Rheumatology, Odense University Hospital, Odense, Denmark; 11 Department of Internal Medicine, Little Belt Hospital Kolding, Kolding, Denmark; 12 Department of Clinical Biochemistry, Odense University Hospital, Odense, Denmark; 13 Department of Cardiothoracic and Vascular Surgery, Odense University Hospital,Odense, Denmark; Institut d'Investigacions Biomediques de Barcelona, SPAIN

## Abstract

**Background and aims:**

Vitamin K antagonists (VKA) remain the most frequently prescribed oral anticoagulants worldwide despite the introduction of non-vitamin K antagonist oral anticoagulants (NOAC). VKA interfere with the regeneration of Vitamin K1 and K2, essential to the activation of coagulation factors and activation of matrix-Gla protein, a strong inhibitor of arterial calcifications. This study aimed to clarify whether VKA treatment was associated with the extent of coronary artery calcification (CAC) in a population with no prior cardiovascular disease (CVD).

**Methods:**

We collected data on cardiovascular risk factors and CAC scores from cardiac CT scans performed as part of clinical examinations (n = 9,672) or research studies (n = 14,166) in the period 2007–2017. Data on use of anticoagulation were obtained from the Danish National Health Service Prescription Database. The association between duration of anticoagulation and categorized CAC score (0, 1–99, 100–399, ≥400) was investigated by ordered logistic regression adjusting for covariates.

**Results:**

The final study population consisted of 17,254 participants with no prior CVD, of whom 1,748 and 1,144 had been treated with VKA or NOAC, respectively. A longer duration of VKA treatment was associated with higher CAC categories. For each year of VKA treatment, the odds of being in a higher CAC category increased (odds ratio (OR) = 1.032, 95%CI 1.009–1.057). In contrast, NOAC treatment duration was not associated with CAC category (OR = 1.002, 95%CI 0.935–1.074). There was no significant interaction between VKA treatment duration and age on CAC category.

**Conclusions:**

Adjusted for cardiovascular risk factors, VKA treatment–contrary to NOAC—was associated to higher CAC category.

## Introduction

Vitamin K antagonists (VKA) have been the most widely prescribed anticoagulants since their introduction in 1954. Around 75,500 Danes (1.3% of the population) were treated with VKA in 2017 [1]. Despite the introduction of non-vitamin K antagonist oral anticoagulants (NOAC), VKA are still the most frequently used anticoagulants worldwide because of its long history, low price and broader therapeutic use compared to NOAC. The prevalence of cardiovascular disease (CVD) is increasing due to the aging population, and the use of anticoagulants is expected to rise [2].

VKA inhibit the recycling of vitamin K, including phylloquinone (vitamin K1) and menaquinone (vitamin K2). They are essential for activation of functional clotting factors II, VII, IX and X, and γ-carboxylation of proteins involved in inhibition of arterial calcification, i.e. matrix-Gla proteins (MGP), respectively. MGP is a potent local inhibitor produced in the vascular smooth muscle cells in the vessel wall. It acts by inhibiting calcium crystal formation and regulating bone morphogenetic protein 2, which is a growth factor responsible for osteogenic differentiation [3]. Arterial calcifications caused by MGP-deficiency were originally shown in MGP-null mice in 1997 [4]. As VKA interferes with the vitamin K-driven γ-carboxylation of MGP, the balance of cellular calcium uptake and the mineralization process in bone and blood vessels is impaired, subsequently promoting vascular calcification [5, 6]. In animal studies, the inhibition of the vitamin K-dependent proteins by VKA resulted in arterial and soft tissue calcification [5, 7]. It has also been suggested that VKA are associated with enhanced tissue calcification including coronary artery calcification (CAC) in humans [6, 8–10]. As CAC is a strong and independent predictor of CVD [11] this may be a truly unwanted side effect of VKA.

Thus, the aim of this study is to clarify if VKA treatment, after adjustment for standard cardiovascular risk factors, is associated with the presence and extent of CAC in a large patient population with no prior cardiovascular disease.

## Materials and methods

### Study design and population

This is a multicenter, observational study. More than 24,000 cardiac computed tomography (CT) scans were performed between 2007 and 2017 as part of clinical examinations at Odense University Hospital, or as part of research studies at five centres (Odense, Svendborg, Vejle, Esbjerg, Silkeborg). After approval from the Danish Data Protection Agency, traditional cardiovascular risk factors and CAC scores were collected from the electronic patient databases at Odense University Hospital, the Western Denmark Heart Registry [12], The National Danish Ablation Database [13], and research databases (DANCAVAS [14], DanRisk [15], IDA [16], MYODAN [17], NOTICE [18], AMFAST [19], and Ilangkovan N et al [20].). The study participants’ medication status since 2004 was collected from the Danish National Health Service Prescription Database (DNHSPD) [21]. For each participant, all prescriptions before the CT scan were identified in data from DNHSPD.

All individuals having a cardiac CT scan performed within the study period were included. Subjects with missing patient ID numbers, examination date or CAC score were excluded. If a participant had a clinical examination as well as a research examination performed, the latter was selected as data on risk factors and CAC score were most complete for these. If more than one CT scan was performed the latest was included ensuring the longest history of VKA exposure. Further, participants below the age of 18 and patients with CVD were excluded.

Baseline characteristics were defined as follows; Diabetes mellitus was defined as known diabetes, HbA1c ≥48.0 mmol/mol, or antidiabetic treatment within three months prior to the CT-scan. Hypertension was defined as known hypertension, measured systolic blood pressure ≥160 mmHg, diastolic blood pressure ≥100 mmHg [14], or antihypertensive treatment within three months prior to the CT-scan. Hypercholesterolemia was defined as known hypercholesterolemia, a total cholesterol ≥5.0 mmol/L, LDL-cholesterol ≥3.0 mmol/L, or treatment with statins within three months prior to the CT-scan. CVD was self-reported and defined as prior myocardial infarction, coronary revascularization, stroke, or peripheral artery disease. Family history of CVD was defined as a first degree relative (men age <55 years and women age <65 years) with history of CVD. The most widely recommended CKD-EPI Creatinine Equation (2009) was used to estimate the glomerular filtration rate (eGFR) [22]. Chronic kidney disease (CKD) was defined as eGFR less than 60 mL/min.

VKA included warfarin (ATC, B01AA03) and phenprocoumon (B01AA04), while NOAC included apixaban (B01AF02), dabigatran (B01AE07), rivaroxaban (B01AF01) and edoxaban (B01AF03). In keeping with our mechanistic understanding of how VKA might affect CAC, our exposure measure was cumulative treatment duration, rather than focusing on ongoing use at the time of CT scan or on cumulative dose. The number of days’ supply for a prescription was not given in our data source. Instead, durations of VKA and NOAC treatment were calculated from the assumption that a user of anticoagulants might have been treated in periods, for which reason treatment episodes were defined. Treatment episodes of VKA and NOAC were built by assigning a treatment period to each prescription. If the treatment period of one prescription covered the starting date of a following prescription on the same drug, then these two prescriptions were thought to belong to the same, uninterrupted episode. Different rules were used for VKA and NOAC. For VKA, a treatment period of a prescription was set to start on the date of dispensing and lasting for 100 days. The 100-day treatment period was tested in a number of sensitivity analyses, ranging from 50 to 150 days. Hereby individual dosage concerns were avoided. Treatment periods of NOAC prescriptions were calculated with the assumption that the user took one tablet per day, two if it was apixaban or dabigatran. Furthermore, a grace period of 30 days was added to each NOAC prescription to account for irregular dispensing due to imperfect adherence or stockpiling, i.e., the total treatment period assigned to a NOAC prescription was the number or half the number of tablets, depending on the drug, plus 30 days. The last prescription in an episode was assigned the same duration as all other prescriptions. The total duration of anticoagulant treatment for an individual was calculated simply by adding the duration of episodes.

The outcome variable, CAC score, was measured in non-contrast CT scans using established software (syngo.CT CaScoring–Siemens Healthcare). CAC score was assessed by summing the scores from all foci in the coronary arteries and expressed in Agatston units (AU) [23]. The analyses were performed by expert physicians and skilled radiographers at the different medical centers.

### Statistics

Participant characteristics are tabulated by the four anticoagulation treatment groups of interest; never users of VKA/NOAC, ever users of VKA, ever users of NOAC, ever users of both VKA and NOAC. Variables are presented as n (%), mean ± standard deviation (SD) or median with 25^th^ and 75^th^ percentiles where appropriate. Empirical histograms were used to evaluate if a continuous variable followed a normal distribution. Means were compared by Student’s t-test (two groups) or one-way ANOVA test (multiple groups), medians by nonparametric k-sample test with continuity correction where available and categorical variables by chi-square test.

Since the CAC score is highly right skewed and with excess zero-values it was categorized for further analyses. The categorisation was based on commonly used cut-points: 0, 1–99, 100–399 and ≥400 AU, corresponding to no, mild, moderate and severe atherosclerotic plaque burden, respectively [24]. The association between the categorized CAC score and VKA was investigated by ordered logistic regression in which all known cardiovascular risk factors and possible confounders were included. The independent variables included in the model were: age, gender, smoking, body mass index (BMI), diabetes mellitus, hypertension, hypercholesterolemia, family history of CVD, eGFR, VKA treatment duration and NOAC treatment duration. Moreover, propensity score adjustment was included in the model in a separate analysis.

As CAC score is associated with age test for interaction effects between age and treatment duration of VKA as well as NOAC on CAC score was performed to assess whether age stratification was necessary.

In all cases, two-tailed p-values <0.05 were considered to be statistically significant. All analyses were performed by Stata statistical software (version 15, StataCorp, College Station, Texas 77845 USA).

## Results

### Baseline characteristics

**Fig 1:**
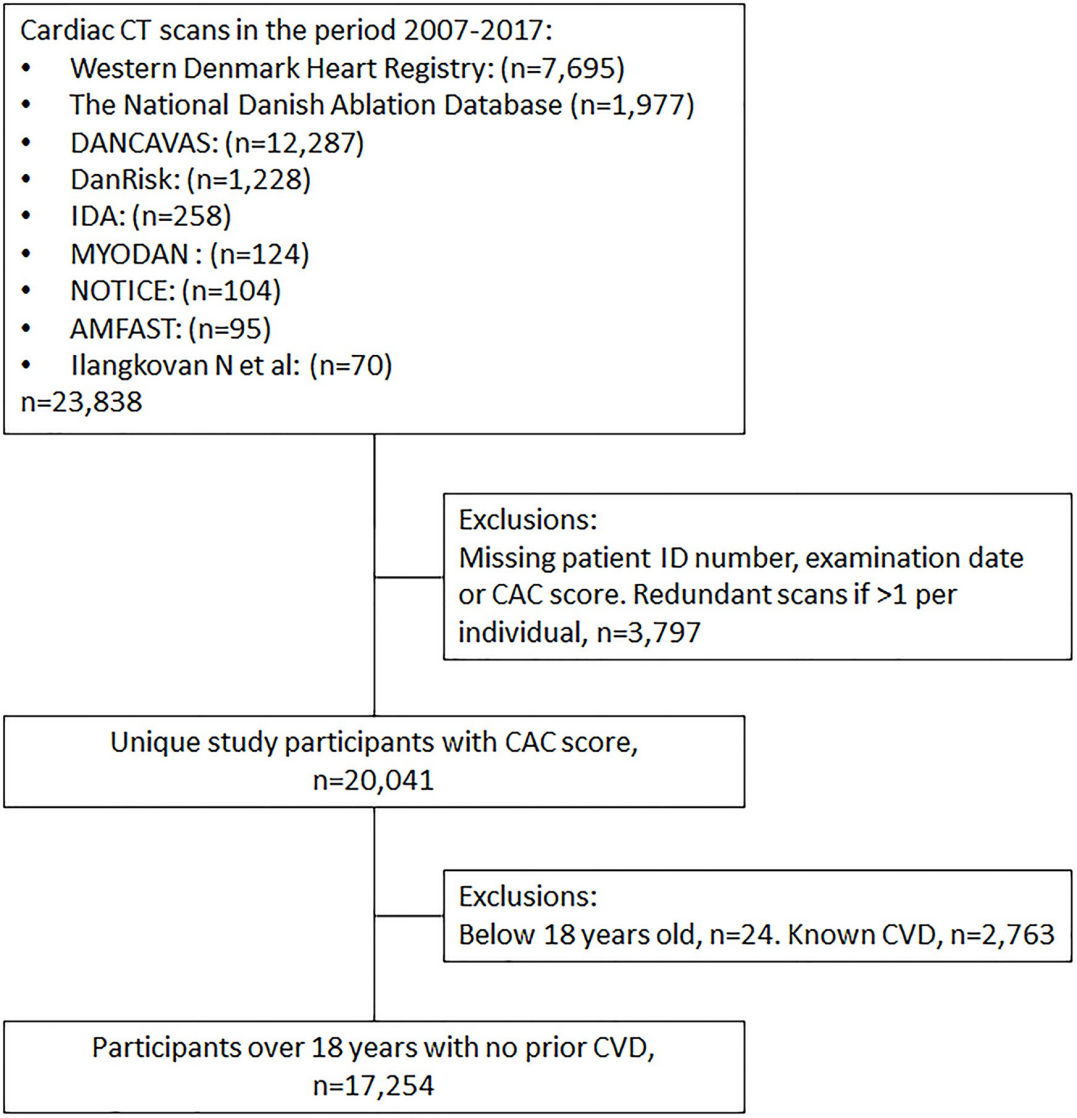
Selection of study population.

In total, data on 23,838 cardiac CT scans were collected. Due to missing data or individuals with several CT scans, 3,797 were excluded. Furthermore, participants below the age of 18 (n = 24) and patients with known CVD (n = 2,763) were excluded. The final study population included 17,254 participants (Fig 1).

**Fig 2:**
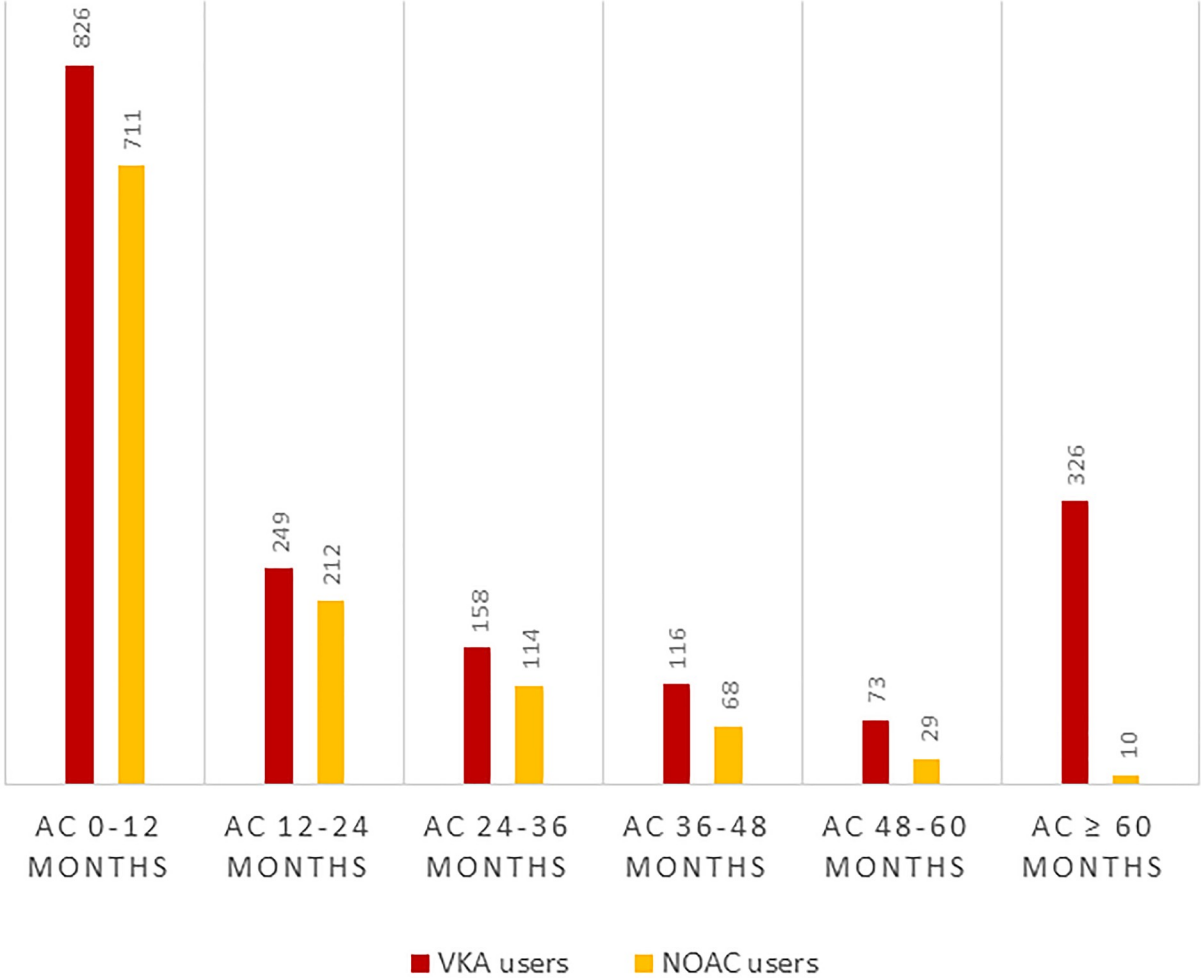
Treatment duration of VKA and NOAC. AC, Anticoagulants.

Table 1 describes the baseline characteristics of the study participants stratified by anticoagulation treatment status. Overall, the participants had a median age of 67 years, and 75% (n = 12,946) were men; 10% (n = 1,748) were ever users of VKA and 7% (n = 1,144) were ever users of NOAC. Apart from long term treatment (≥5years), the treatment duration of VKA and NOAC was similar (Fig 2). For patients treated with anticoagulants, the median treatment durations were 13.8 and 8.0 months with VKA and NOAC, respectively. When subjects treated exclusively with either VKA (6%, n = 1,064) or NOAC (3%, n = 460) were contrasted in a crude comparison, the VKA group had a smaller proportion of men (p = 0.004), were younger (p<0.001), and had a lower median CAC score (48 versus 83, p = 0.02), but did not differ significantly in any other parameters. By comparing the median CAC score in each of the three treated groups with the non-treated group, the ever users of NOAC had a significantly higher CAC score (p = 0.002), while VKA users and users of both VKA and NOAC did not differ from the never users (p = 0.39, p = 0.79).

**Table 1:** Participant characteristics by anticoagulation treatment.

Characteristics	All subjects	Never users of VKA/NOAC	Ever users of VKA, but not NOAC	Ever users of NOAC, but not VKA	Ever users of both VKA and NOAC	p-value^a^	p-value^b^
	(n = 17,254)	(n = 15,046, 87%)	(n = 1,064, 6%)	(n = 460, 3%)	(n = 684, 4%)		
Male	12,946 (75)	11,267 (75)	806 (76)	379 (82)	494(72)	0.001	0.004
Age, yrs	67 (61, 70.4)	66.9 (61.3, 70.3)	67.1 (58.7, 71.4)	69.0 (64.2, 71.8)	66.0 (59.2, 70.6)	<0.001	<0.001
Body mass index, kg/m^2^	27.5 (± 4.6)	27.4 (±4.5)	28.4(± 5.2)	28.8 (± 5.3)	28.2 (±5.3)	<0.0001	0.13
Diabetes	1,836 (11)	1,586 (11)	136 (13)	62 (13)	52 (8)	0.001	0.71
Hypertension	10,276 (60)	8,398 (56)	879 (83)	390 (85)	609 (89)	<0.001	0.3
Hypercholesterolemia	11,035 (64)	10,015 (67)	532 (50)	255 (55)	233 (34)	<0.001	0.05
Statins	4,242 (25)	3,584 (24)	347 (33)	127(28)	184 (27)	<0.001	0.05
Atrial fibrillation/flutter	1,781 (10)	196 (1)	698 (66)	278 (60)	609 (89)	<0.001	0.002
Smoking status						<0.001	0.96
*Non-smokers*	6,565 (38)	5,671 (38)	441 (41)	192 (42)	261 (38)		
*Former smokers*	7,720 (45)	6,675 (44)	498 (47)	211 (46)	336 (49)		
*Active smokers*	2,869 (17)	2,626 (17)	114 (11)	51 (11)	78 (11)		
Family history of CVD	3,463 (21)	2,994 (20)	227 (22)	90 (20)	152 (24)	0.03	0.63
HDL, mmol/L	1.4(1.2, 1.7)	1.4 (1.2, 1.7)	1.3 (1.1, 1.6)	1.4 (1.1, 1.6)	1.4 (1.2, 1.6)	<0.001	0.18
LDL, mmol/L	3.1 (± 0.9)	3.1(±0.9)	2.8(±1.1)	2.8 (±0.9)	2.7 (±0.9)	<0.0001	0.93
Total cholesterol, mmol/L	5.2 (± 1.1)	5.2 (±1.0)	4.9(± 1.2)	5.0 (±1.1)	4.8(±1.0)	<0.0001	0.92
Creatinine, μmol/L	84 (± 27)	83 (±25)	93 (±50)	89 (±19)	88 (±19)	<0.0001	0.12
eGFR, mL/min	80 (± 15)	81 (±15)	76 (±18)	75(±16)	76 (±15)	<0.0001	0.88
Chronic kidney disease, eGFR < 60 mL/min	1,544 (9)	1,204 (8)	171 (16)	76 (17)	93 (14)	<0.001	0.8
Systolic blood pressure, mmHg	147 (± 20)	147 (±20)	146 (± 21)	146(±21)	147 (± 23)	0.09	0.91
Diastolic blood pressure, mmHg	83(± 11)	82(± 11)	84(±12)	84(±12)	88(±14)	<0.0001	0.27
CAC score, AU	40 (0, 261)	40 (0, 249)	48 (0, 339)	83 (1, 418)	35 (0, 325)	0.01	0.02
*0*	5,253 (30)	4,628 (31)	310 (29)	114 (25)	201 (29)		
*1–99*	5,264 (31)	4,612 (31)	319 (30)	125 (27)	208 (31)		
*100–399*	3,473 (20)	3,031 (20)	206 (19)	105 (23)	131 (19)		
*≥400*	3,264 (19)	2,775 (18)	229 (22)	116 (25)	144 (21)		

Abbreviations: AU, Agatston units; CAC, coronary artery calcification; CVD, cardiovascular disease; eGFR, estimated glomerular filtration rate; HDL, high-density lipoprotein; LDL, low-density lipoprotein; NOAC, non-vitamin K antagonist oral anticoagulants; VKA, vitamin K antagonists.

^a^p-value for difference between all treatment groups.

^b^p-value for difference between ever users of VKA alone and ever users of NOAC alone.

Values are n (%), mean (±SD) or median (25^th^ percentile, 75^th^ percentile). Two-sided p-values are shown for categorical data compared by chi-square tests; means were compared by one-way ANOVA tests, medians by nonparametric k-sample tests with continuity correction.

### Anticoagulation treatment and CAC score

**Fig 3:**
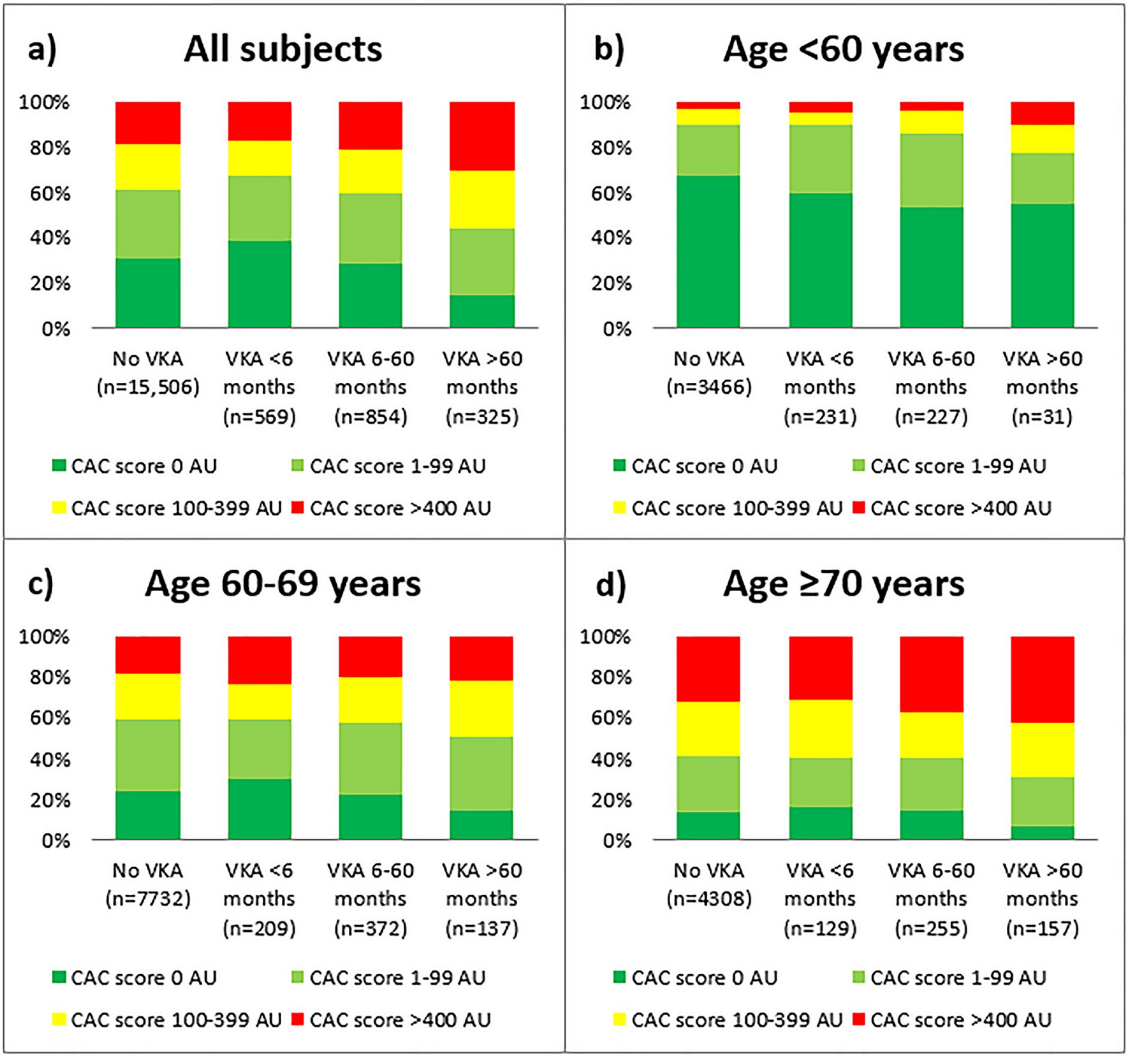
Distribution of coronary artery calcium (CAC) score categories in participants with various durations of VKA treatment according to all subjects (A) and three age groups: (B) age <60 years, (C) age 60–69 years and (D) age ≥70 years. P-values for CAC score category distributions by VKA treatment duration were calculated by chi-square test: A) p<0.001, B) p<0.001, C) p = 0.047 and D) p = 0.064.

Fig 3 shows the distribution of the different CAC score categories in patients with various durations of VKA treatment according to age groups. By chi-square test, the distribution was significantly different according to the duration of VKA use (p<0.001) in the pooled cohort. Significant difference was also found in patients aged <60 years and 60–69 years with p<0.001 and p = 0.047, respectively, while the difference was on the border of significance (p = 0.064) in patients with the age ≥70 years.

Table 2 shows the output of the ordered logistic regression performed on the entire population including all non-treated and treated participants for which the full profile was available (n = 15,958). For each year of VKA treatment the odds of being in a higher CAC category, i.e. having more severe CAC, increased by 3.2% (odds ratio (OR) = 1.032, 95%CI 1.009–1.057). Traditional risk factors such as male gender, age, smoking, BMI, diabetes, hypertension, dyslipidemia, family history of CVD and eGFR were also positively associated with CAC category. NOAC treatment was not found to be significantly associated with CAC category (OR = 1.002, 95%CI 0.935–1.074). By dichotomizing the CAC score (CAC = 0 if CAC score = 0 and CAC = 1 if CAC score>0) it was demonstrated that two years of VKA treatment is equal to being one year older (OR = 1.052, 95%CI 1.013–1.093 versus OR = 1.110, 95%CI 1.104–1.117), S1 Table. Short-term treatment with VKA for less than 6 months was not associated with CAC category (OR = 1.315, 95%CI 0.633–2.732). In a supplementary analysis of patients without statin treatment (n = 12,143), the association between VKA treatment duration and CAC category was unchanged (OR = 1.036, 95%CI 1.006–1.067), S2 Table. Additional inclusion of biomarkers like serum Calcium / Phosphate (n = 10,922) and CRP (n = 10,070) did not change the association (OR = 1.038, 95%CI 1.008–1.070 and OR = 1.033, 95%CI 1.002–1.064, respectively), S3 and S4 Tables. The results were consistent after propensity score adjustment (OR = 1.033, 95%CI 1.010–1.057), S5 Table.

**Table 2:** Multivariate ordered logistic regression model of the association between duration of VKA treatment and coronary artery calcification. 15,958 subjects with a full profile were included in the analysis.

	CAC score^a^ (outcome variable)		
*Predictor variable*	*OR*	*95% CI*	*p-value*
Age, yrs	1.116	1.111–1.122	<0.001
Sex			
*Male*	3.095	2.858–3.351	<0.001
Smoking status			
*Former smoker*	1.387	1.299–1.482	<0.001
*Active smoker*	2.155	1.970–2.358	<0.001
BMI, kg/m^2^	1.014	1.007–1.021	<0.001
Diabetes	1.883	1.702–2.083	<0.001
Hypertension	1.788	1.677–1.907	<0.001
Hypercholesterolemia	1.445	1.351–1.545	<0.001
Family history of CVD	1.429	1.320–1.546	<0.001
eGFR, mL/min	1.006	1.004–1.008	<0.001
VKA, yrs	1.032	1.009–1.057	0.007
NOAC, yrs	1.002	0.935–1.074	0.96

Abbreviations: BMI, body mass index; CAC, coronary artery calcification; CI, confidence interval; CVD, cardiovascular disease; eGFR, estimated glomerular filtration rate; NOAC, non-vitamin K antagonist oral anticoagulants; OR, odds ratio; VKA, vitamin K antagonists.

^a^CAC score is divided into following 4 categories: 0, 1–99, 100–399, ≥400 Agatston units.

Sensitivity analysis regarding treatment period assigned to each VKA prescription showed our results to be robust (50 days: OR = 1.036 (95%CI 1.006–1.067), and 150 days OR = 1.031 (95%CI 1.009–1.054). In another sensitivity analysis (zero-inflated negative binomial regression), VKA had a borderline significant effect on CAC score, S6 Table. One year of VKA treatment increased the odds of being one Agatston Unit higher with 2.1% (IRR = 1.0206, 95%CI 0.9995–1.0421). Also, in this analysis NOAC treatment was not associated with CAC score (IRR = 1.037, 95%CI 0.974–1.105).

Test for interaction effects between age and duration of VKA treatment as well as duration of NOAC treatment showed no significant interaction effect of VKA on CAC score in any age group, S7 Table.

## Discussion

In this retrospective observational multicenter study on Danish men and women with no prior CVD we made an essential discovery. Independently of age and other traditional cardiovascular risk factors, the duration of VKA treatment was significantly associated with the presence and extent of CAC. Of importance, duration of NOAC treatment had no association with CAC. This despite the fact that VKA users had a more benign risk profile considering their lower median CAC score, lower age at the time of the CT scan and smaller proportion of men compared to the NOAC users.

A number of imaging modalities are available for detecting artery calcifications. In this study the CAC score was assessed by cardiac CT, which is an important tool for identifying patients at increased risk of CVD [25]. Yet, this method is not able to give the exact localization of the calcium deposits in the coronary arteries and hereby classify the calcification as either intimal or medial. A mouse model showed that absence of MGP leads to calcification of the medial and especially the intimal layer [9]. Distinction between the two calcification types is important as intima calcification is a risk factor for plaque rupture and has the highest risk of CVD [26]. A meta-analysis among 30 prospective studies found that presence of calcification in any artery wall is associated with a 3–4 fold higher risk for mortality and cardiovascular events [27]. However, a detailed insight into the true impact of VKA on CAC and plaque vulnerability remains to be obtained.

Age is a major cardiovascular risk factor, and thus, not surprisingly, age was found to be independently associated with CAC score in the present study. To clarify if age was a confounder causing a spurious association between the CAC score and duration of VKA use, we performed an interaction analysis. Importantly, no interaction effect was found. Thus, the participants were not stratified by age in our analyses, but as shown in Fig 3, CAC score increased with both increasing age and increasing duration of VKA use in all age groups. Importantly, we found that short-term use of VKA (less than 6 months) was not associated with increased risk.

Consistent with the results of the present study, prior imaging studies have shown the procalcific effect of VKA in various vascular beds. In a post hoc randomized trial warfarin use was associated with progressive coronary calcification in patients with coronary artery disease (CAD) evaluated by serial coronary intravascular ultrasound examinations [10]. Another study on patients with suspected CAD, who underwent CT, also showed that VKA treatment was associated with coronary artery plaque calcification [9]. Likewise, a prospective study on patients with AF and no prior CVD showed an association between VKA use and levels of coronary calcification measured by cardiac CT [8]. The impact of long-term VKA treatment was investigated in a case-control study, which showed that VKA leads to CAC in a time-dependent matter, as it has been suggested in this present study [28]. Two recent studies similarly to the current compared CAC evaluated by CT in patients treated with VKA or NOAC. These smaller studies had consistent results showing an association between VKA and calcification, while maybe even a beneficial effect of NOAC on atherosclerosis [29, 30].

Thus, there are a number of smaller studies showing an association between VKA and CAC. To extend our knowledge, prospective studies and randomized controlled trials with a wide range of patients would be advantageous. A recent prospective, randomized, open-label study compared the progression in coronary plaque in patients treated with either warfarin or rivaroxaban. With a 1-year follow-up, warfarin was found to be significantly associated with progression of total plaque volume after adjustment for cardiovascular risk factors [31]. Now an on-going double-blinded, randomized, placebo-controlled trial investigates the difference in progression of CAC score in patients with CAD randomized to either vitamin K2 supplementation or placebo [32]. A similar on-going study seeks to examine if vitamin K2 supplementation can slow down the calcification of aortic valves in patients with substantial aortic valve calcification [33]. These studies will hopefully add knowledge on the importance of vitamin K in tissue calcification.

The findings of the present and prior studies suggest that long-time use of VKA, together with other risk factors, may enhance the progression of CAC significantly. NOAC do not interfere with the vitamin K cascade, and were not associated with higher levels of CAC score. Considering the aging population and the associated increased use of anticoagulant therapy in patients with an increased thromboembolic risk, the observations in this study may have scientific and pharmacologic interest.

### Study limitations and strengths

A major limitation to our study is the lack of hard cardiovascular endpoints, which prevents us from learning the true consequence of the higher CAC score by long-term VKA treatment. The study is also limited by its observational design, why the cause and effect relationship between VKA and CAC score might be open to interpretation. Furthermore, the medication status was only evaluated since 2004, while it would have been advantageous to have a life-long description of the VKA and NOAC use for a more accurate analysis. As NOAC is a relatively new medical treatment, fewer patients were in long NOAC treatments and this might affect our results.

However, in comparison with previous studies, this study has the largest sample size to date. Due to the extensive numbers of descriptive data variables and a very large number of participants we were able to adjust for traditional cardiovascular risk factors and use of medical treatment. Data on risk factors as well as medication status were collected from databases and registries with high validity [21]. Moreover, this study is the largest to investigate the effect of both VKA and NOAC treatment duration on CAC, which minimizes the risk of confounding by indication for the association between VKA and CAC. Another strength is that the participants were free of CVD, which reduced the impact of associated vascular disease on CAC, thereby enhancing identification of potentially harmful effects of VKA.

## Conclusions

Adjusted for cardiovascular risk factors, duration of VKA treatment, but not NOAC treatment, was associated with the risk of a higher level of CAC score in adults with no prior CVD. The procalcific effect of VKA is consistent with the findings in earlier imaging studies.

## Supporting information

S1 Table Dichotomized CAC score (CAC = 0 if CAC score = 0 and CAC = 1 if CAC score>0) as outcome variable. Multivariate ordered logistic regression model of the association between duration of VKA treatment and coronary artery calcification. 15,958 subjects with a full profile were included in the analysis.
(DOCX)

S2 Table Exclusion of patients in statin treatment. Multivariate ordered logistic regression model of the association between duration of VKA treatment and coronary artery calcification. 12,143 subjects with a full profile were included in the analysis.
(DOCX)

S3 Table Inclusion of serum calcium and phosphate in the model. Multivariate ordered logistic regression model of the association between duration of VKA treatment and coronary artery calcification. 10,922 subjects with a full profile were included in the analysis.
(DOCX)

S4 Table Inclusion of CRP in the model. Multivariate ordered logistic regression model of the association between duration of VKA treatment and coronary artery calcification. 10,070 subjects with a full profile were included in the analysis.
(DOCX)

S5 Table Inclusion of propensity score adjustment in the model. Propensity score adjustment in an ordered logistic regression model of the association between duration of VKA treatment and coronary artery calcification. 15,958 subjects with a full profile were included in the analysis.
(DOCX)

S6 Table Sensitivity analysis using zero-inflated negative binomial regression. Zero-inflated negative binominal regression model of the association between duration of VKA treatment and coronary artery calcification. 15,958 subjects with a full profile were included in the analysis.
(DOCX)

S7 Table Interaction analysis in the ordered logistic regression model of the association between duration of anticoagulation treatment and coronary artery calcification. (DOCX)

S1 File Declaration of interests. (DOCX)

S2 File Statement of originality. (DOCX)
