# Supplementary material for: Extent of arterial calcification by conventional vitamin K antagonist treatment
Source: PLoS One. 2020 Oct 29;15(10):e0241450. doi: 10.1371/journal.pone.0241450 (PMC7595268; doi:10.1371/journal.pone.0241450)
Supplement: S1 Table — Multivariate ordered logistic regression model of the association between duration of VKA treatment and coronary artery calcification. 15,958 subjects with a full profile were included in the analysis. (DOCX) [file pone.0241450.s001.docx]

| **S1 Table** | | | |
| --- | --- | --- | --- |
|  | **CAC score^a^ (outcome variable)** | | |
| ***Predictor variable*** | ***OR*** | ***95% CI*** | ***p-value*** |
| Age, yrs | 1.110 | 1.104-1.117 | <0.001 |
| *Male* | 3.206 | 2.924-3.516 | <0.001 |
| Smoking status  *Former smoker  Active smoker* | 1.422  2.205 | 1.302-1.552  1.947-2.498 | <0.001  <0.001 |
| BMI, kg/m^2^ | 1.018 | 1.008-1.028 | <0.001 |
| Diabetes | 1.767 | 1.501-2.080 | <0.001 |
| Hypertension | 1.623 | 1.491-1.766 | <0.001 |
| Hypercholesterolemia | 1.563 | 1.433-1.705 | <0.001 |
| Family history of CVD | 1.402 | 1.262-1.557 | <0.001 |
| eGFR, mL/min | 1.006 | 1.002-1.009 | <0.001 |
| VKA, yrs | 1.052 | 1.013-1.093 | 0.009 |
| NOAC, yrs | 0.987 | 0.892-1.092 | 0.796 |
| Abbreviations: BMI, body mass index; CAC, coronary artery calcification; CI, confidence interval; CVD, cardiovascular disease; eGFR, estimated glomerular filtration rate; NOAC, non-vitamin K antagonist oral anticoagulants; OR, odds ratio; VKA, vitamin K antagonists.  ^a^CAC score is divided into 2 categories: 0 or >0 Agatston Units. | | | |
