# Supplementary material for: Extent of arterial calcification by conventional vitamin K antagonist treatment
Source: PLoS One. 2020 Oct 29;15(10):e0241450. doi: 10.1371/journal.pone.0241450 (PMC7595268; doi:10.1371/journal.pone.0241450)
Supplement: S2 Table — Multivariate ordered logistic regression model of the association between duration of VKA treatment and coronary artery calcification. 12,143 subjects with a full profile were included in the analysis. (DOCX) [file pone.0241450.s002.docx]

| **S2 Table** | | | |
| --- | --- | --- | --- |
|  | **CAC score^a^ (outcome variable)** | | |
| ***Predictor variable*** | ***OR*** | ***95% CI*** | ***p-value*** |
| Age, yrs | 1.116 | 1.110-1.122 | <0.001 |
| *Male* | 3.286 | 2.987-3.616 | <0.001 |
| Smoking status  *Former smoker  Active smoker* | 1.352  2.272 | 1.253-1.458  2.049-2.520 | <0.001  <0.001 |
| BMI, kg/m^2^ | 1.017 | 1.008-1.025 | <0.001 |
| Diabetes | 1.819 | 1.562-2.118 | <0.001 |
| Hypertension | 1.605 | 1.493-1.725 | <0.001 |
| Hypercholesterolemia | 1.224 | 1.136-1.320 | <0.001 |
| Family history of CVD | 1.364 | 1.241-1.499 | <0.001 |
| eGFR, mL/min | 1.007 | 1.004-1.010 | <0.001 |
| VKA, yrs | 1.036 | 1.006-1.067 | 0.018 |
| NOAC, yrs | 1.030 | 0.946-1.121 | 0.501 |
| Abbreviations: BMI, body mass index; CAC, coronary artery calcification; CI, confidence interval; CVD, cardiovascular disease; eGFR, estimated glomerular filtration rate; NOAC, non-vitamin K antagonist oral anticoagulants; OR, odds ratio; VKA, vitamin K antagonists.  ^a^CAC score is divided into following 4 categories: 0, 1-99, 100-399, ≥400 Agatston Units. | | | |
