# Supplementary material for: Extent of arterial calcification by conventional vitamin K antagonist treatment
Source: PLoS One. 2020 Oct 29;15(10):e0241450. doi: 10.1371/journal.pone.0241450 (PMC7595268; doi:10.1371/journal.pone.0241450)
Supplement: S3 Table — Multivariate ordered logistic regression model of the association between duration of VKA treatment and coronary artery calcification. 10,922 subjects with a full profile were included in the analysis. (DOCX) [file pone.0241450.s003.docx]

| **S3 Table** | | | |
| --- | --- | --- | --- |
|  | **CAC score^a^ (outcome variable)** | | |
| ***Predictor variable*** | ***OR*** | ***95% CI*** | ***p-value*** |
| Age, yrs | 1.115 | 1.104-1.125 | <0.001 |
| *Male* | 3.980 | 3.492-4.536 | <0.001 |
| Smoking status  *Former smoker  Active smoker* | 1.354  2.015 | 1.254-1.462  1.809-2.345 | <0.001  <0.001 |
| BMI, kg/m^2^ | 1.023 | 1.014-1.032 | <0.001 |
| Diabetes | 1.842 | 1.635-2.074 | <0.001 |
| Hypertension | 1.836 | 1.703-1.979 | <0.001 |
| Hypercholesterolemia | 1.344 | 1.227-1.471 | <0.001 |
| Family history of CVD | 1.506 | 1.356-1.672 | <0.001 |
| eGFR, mL/min | 1.007 | 1.005-1.010 | <0.001 |
| Calcium, mmol/L | 2.284 | 1.532-3.405 | <0.001 |
| Phosphate, mmol/L | 3.409 | 2.730-4.258 | <0.001 |
| VKA, yrs | 1.038 | 1.008-1.070 | 0.014 |
| NOAC, yrs | 1.053 | 0.949-1.169 | 0.33 |
| Abbreviations: BMI, body mass index; CAC, coronary artery calcification; CI, confidence interval; CVD, cardiovascular disease; eGFR, estimated glomerular filtration rate; NOAC, non-vitamin K antagonist oral anticoagulants; OR, odds ratio; VKA, vitamin K antagonists.  ^a^CAC score is divided into following 4 categories: 0, 1-99, 100-399, ≥400 Agatston Units. | | | |
