# Supplementary material for: Extent of arterial calcification by conventional vitamin K antagonist treatment
Source: PLoS One. 2020 Oct 29;15(10):e0241450. doi: 10.1371/journal.pone.0241450 (PMC7595268; doi:10.1371/journal.pone.0241450)
Supplement: S4 Table — Multivariate ordered logistic regression model of the association between duration of VKA treatment and coronary artery calcification. 10,070 subjects with a full profile were included in the analysis. (DOCX) [file pone.0241450.s004.docx]

| **S4 Table** | | | |
| --- | --- | --- | --- |
|  | **CAC score^a^ (outcome variable)** | | |
| ***Predictor variable*** | ***OR*** | ***95% CI*** | ***p-value*** |
| Age, yrs | 1.128 | 1.115-1.141 | <0.001 |
| *Male* | 3.567 | 3.059-4.159 | <0.001 |
| Smoking status  *Former smoker  Active smoker* | 1.366  2.116 | 1.261-1.479  1.890-2.369 | <0.001  <0.001 |
| BMI, kg/m^2^ | 1.023 | 1.013-1.032 | <0.001 |
| Diabetes | 1.945 | 1.723-2.195 | <0.001 |
| Hypertension | 1.831 | 1.695-1.978 | <0.001 |
| Hypercholesterolemia | 1.377 | 1.255-1.511 | <0.001 |
| Family history of CVD | 1.459 | 1.306-1.629 | <0.001 |
| eGFR, mL/min | 1.006 | 1.004-1.009 | <0.001 |
| CRP, mg/L | 1.002 | 0.997-1.007 | 0.38 |
| VKA, yrs | 1.033 | 1.002-1.064 | 0.034 |
| NOAC, yrs | 1.053 | 0.948-1.168 | 0.34 |
| Abbreviations: BMI, body mass index; CAC, coronary artery calcification; CI, confidence interval; CVD, cardiovascular disease; eGFR, estimated glomerular filtration rate; NOAC, non-vitamin K antagonist oral anticoagulants; OR, odds ratio; VKA, vitamin K antagonists.  ^a^CAC score is divided into following 4 categories: 0, 1-99, 100-399, ≥400 Agatston Units. | | | |
