# Supplementary material for: Extent of arterial calcification by conventional vitamin K antagonist treatment
Source: PLoS One. 2020 Oct 29;15(10):e0241450. doi: 10.1371/journal.pone.0241450 (PMC7595268; doi:10.1371/journal.pone.0241450)
Supplement: S5 Table — Propensity score adjustment in an ordered logistic regression model of the association between duration of VKA treatment and coronary artery calcification. 15,958 subjects with a full profile were included in the analysis. (DOCX) [file pone.0241450.s005.docx]

| **S5 Table** | | | |
| --- | --- | --- | --- |
|  | **CAC score^a^ (outcome variable)** | | |
| ***Predictor variable*** | ***OR*** | ***95% CI*** | ***p-value*** |
| Age, yrs | 1.116 | 1.110-1.121 | <0.001 |
| *Male* | 3.158 | 2.910-3.428 | <0.001 |
| Smoking status  *Former smoker  Active smoker* | 1.381  2.095 | 1.293-1.475  1.909-2.302 | <0.001  <0.001 |
| BMI, kg/m^2^ | 1.017 | 1.010-1.025 | <0.001 |
| Diabetes | 1.859 | 1.679-2.058 | <0.001 |
| Hypertension | 2.020 | 1.774-2.301 | <0.001 |
| Hypercholesterolemia | 1.275 | 1.115-1.458 | <0.001 |
| Family history of CVD | 1.439 | 1.330-1.558 | <0.001 |
| eGFR, mL/min | 1.004 | 1.001-1.007 | 0.017 |
| Probability of anticoagulant treatment | 0.351 | 0.133-0.929 | 0.035 |
| VKA, yrs | 1.033 | 1.010-1.057 | 0.006 |
| NOAC, yrs | 1.007 | 0.939-1.079 | 0.843 |
| Abbreviations: BMI, body mass index; CAC, coronary artery calcification; CI, confidence interval; CVD, cardiovascular disease; eGFR, estimated glomerular filtration rate; NOAC, non-vitamin K antagonist oral anticoagulants; OR, odds ratio; VKA, vitamin K antagonists.  ^a^CAC score is divided into following 4 categories: 0, 1-99, 100-399, ≥400 Agatston Units. | | | |
