# Supplementary material for: Extent of arterial calcification by conventional vitamin K antagonist treatment
Source: PLoS One. 2020 Oct 29;15(10):e0241450. doi: 10.1371/journal.pone.0241450 (PMC7595268; doi:10.1371/journal.pone.0241450)
Supplement: S6 Table — Zero-inflated negative binominal regression model of the association between duration of VKA treatment and coronary artery calcification. 15,958 subjects with a full profile were included in the analysis. (DOCX) [file pone.0241450.s006.docx]

| **S6 Table** | | | |
| --- | --- | --- | --- |
|  | **CAC score^a^ (outcome variable)** | | |
| ***Predictor variable*** | ***IRR*** | ***95% CI*** | ***p-value*** |
| Age, yrs | 1.072 | 1.067-1.077 | <0.001 |
| *Male* | 2.024 | 1.865-2.197 | <0.001 |
| Smoking status  *Former smoker  Active smoker* | 1.188  1.662 | 1.116-1.265  1.528-1.807 | <0.001  <0.001 |
| BMI, kg/m^2^ | 1.007 | 1.001-1.014 | 0.03 |
| Diabetes | 1.609 | 1.472-1.759 | <0.001 |
| Hypertension | 1.662 | 1.564-1.766 | <0.001 |
| Hypercholesterolemia | 1.123 | 1.053-1.198 | <0.001 |
| Family history of CVD | 1.352 | 1.254-1.458 | <0.001 |
| eGFR, mL/min | 0.999 | 0.998-1.001 | 0.58 |
| VKA, yrs | 1.0206 | 0.9995-1.0421 | 0.055 |
| NOAC, yrs | 1.037 | 0.974-1.105 | 0.26 |
| Abbreviations: BMI, body mass index; CAC, coronary artery calcification; CI, confidence interval; CVD, cardiovascular disease; eGFR, estimated glomerular filtration rate; IRR, incidence rate ratio; NOAC, non-vitamin K antagonist oral anticoagulants; VKA, vitamin K antagonists.  ^a^CAC score as a count variable (Agatston units, AU). | | | |
