# Supplementary material for: Extent of arterial calcification by conventional vitamin K antagonist treatment
Source: PLoS One. 2020 Oct 29;15(10):e0241450. doi: 10.1371/journal.pone.0241450 (PMC7595268; doi:10.1371/journal.pone.0241450)
Supplement: S7 Table — (DOCX) [file pone.0241450.s007.docx]

| **S7 Table** | | | |
| --- | --- | --- | --- |
|  | **OR** | **95% CI** | **p-value** |
| 61y-67y#VKA | 1.07 | 0.74-1.56 | 0.72 |
| 67-70.4y#VKA | 1.11 | 0.76-1.62 | 0.60 |
| >70.4y#VKA | 1.20 | 0.84-1.71 | 0.31 |
| 61y-67y#NOAC | 0.41 | 0.22-0.76 | 0.004 |
| 67-70.4y#NOAC | 0.76 | 0.42-1.39 | 0.38 |
| >70.4y#NOAC | 1.45 | 0.83-2.54 | 0.19 |
| 61y-67y#Both | 0.71 | 0.45-1.11 | 0.14 |
| 67-70.4y#Both | 0.97 | 0.62-1.51 | 0.88 |
| >70.4y#Both | 1.13 | 0.74-1.73 | 0.56 |
| Abbreviations: CI, confidence interval; NOAC, non-vitamin K antagonist oral anticoagulants; OR, odds ratio; VKA, vitamin K antagonists. | | | |
