# Supplementary material for: Extent of arterial calcification by conventional vitamin K antagonist treatment
Source: PLoS One. 2020 Oct 29;15(10):e0241450. doi: 10.1371/journal.pone.0241450 (PMC7595268; doi:10.1371/journal.pone.0241450)
Supplement: S2 File — (DOCX) [file pone.0241450.s009.docx]

# **Statement of originality**

The manuscript is original work not previously published in any substantial part and is not under consideration of publication elsewhere. The manuscript has been read and approved for submission by all authors.
